# Large-Scale Docking in the Cloud

**DOI:** 10.1021/acs.jcim.3c00031

**Published:** 2023-04-18

**Authors:** Benjamin I. Tingle, John J. Irwin

**Affiliations:** Department of Pharmaceutical Chemistry, University of California San Francisco, 1700 4th Street, MC 2550, San Francisco, California 94158-2330, United States

## Abstract

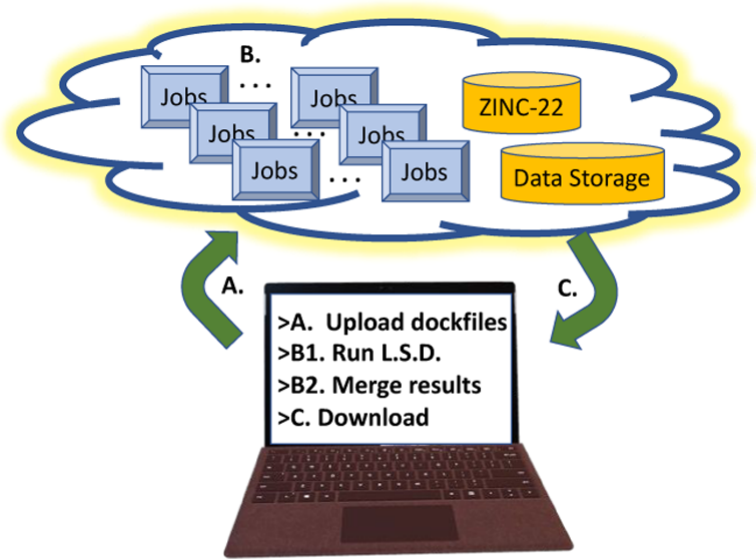

Molecular docking is a pragmatic approach to exploit protein structures for new ligand discovery, but the growing size of available chemical space is increasingly challenging to screen on in-house computer clusters. We have therefore developed AWS-DOCK, a protocol for running UCSF DOCK in the AWS cloud. Our approach leverages the low cost and scalability of cloud resources combined with a low-molecule-cost docking engine to screen billions of molecules efficiently. We benchmarked our system by screening 50 million HAC 22 molecules against the DRD4 receptor with an average CPU time of around 1 s per molecule. We saw up to 3-fold variations in cost between AWS availability zones. Docking 4.5 billion lead-like molecules, a 7 week calculation on our 1000-core lab cluster, runs in about a week depending on accessible CPUs, in AWS for around $25,000, less than the cost of two new nodes. The cloud docking protocol is described in easy-to-follow steps and may be sufficiently general to be used for other docking programs. All the tools to enable AWS-DOCK are available free to everyone, while DOCK 3.8 is free for academic research.

## Introduction

Molecular docking is a pragmatic approach to leverage structures for ligand discovery. The technique screens large libraries, rapidly scoring molecules for fit and ranking the database from best to worst for purchase and testing. Since 2015, the number of commercially accessible compounds has grown by over 3 orders of magnitude, leading to new opportunities to discover new chemistry for new biology. Ultralarge-scale docking (LSD) has now been applied to over a dozen targets^1−11^ and has discovered new compounds with activities often in the nanomolar and occasionally in the sub-nanomolar range,^1,3,6,12^ often leading to molecules with interesting in vivo activities.^3,6,12,13^ A challenge to making this approach widely accessible has been the very size of the new libraries, where over 40 billion tangible molecules have been enumerated, and over 4.5 billion of these have structures calculated that are suitable for docking.

The cost of docking calculations for any target is roughly linear in the number of molecules docked and is embarrassingly parallel, in that it can be spread across multiple processors without meaningful loss in efficiency. Still, whereas a screen of 138 million molecules against the DRD4 receptor^1^ took less than 2 days on our 1000-core cluster, a screen of 4.5 billion molecules would take over 2 months; to finish in under 2 weeks would demand more resources than can often be mustered locally. As the database scales to 10 billion and beyond in the coming years, this capacity problem to obtain timely results will become only more pressing.

**Figure 1:**
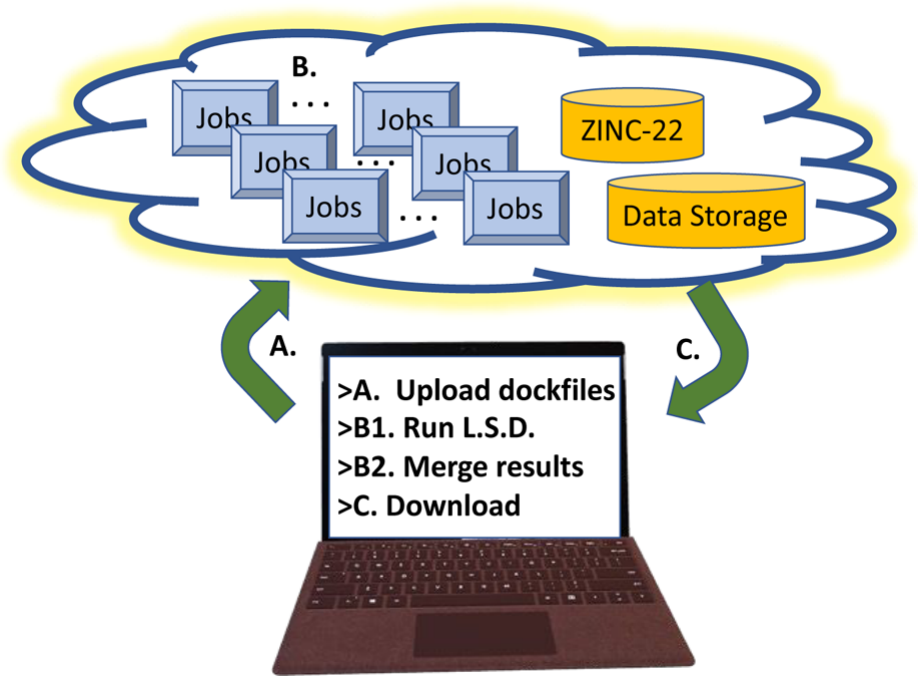
Docking in the cloud. Though conceptually simple, cloud docking presents operational complexities that must be overcome. (A) Upload receptor model and software. The dockable database is preloaded. (B) LSD jobs are run, potentially at an immense scale, and the results are merged to top-hit collections (T.H.C.) within cloud storage. (C) The T.H.C. results are downloaded for prioritization and purchase.

Amazon Web Services (AWS) is one of several cloud providers with the capacity to bring enormous resources to bear on docking screens, if it can be done economically and if the system can be mastered. Though in principle AWS, and related systems, are simply enormous fee-for-use clusters accessible to all (Figure 1), there are intricacies with their efficient use that are barriers to entry for the general user, and it was not certain to us that even when used efficiently, these off-site resources could compete with an on-site academic or industrial cluster or that a protocol suitable for occasional users and nonexperts could be developed.

Regarding cloud-based docking, there have been several studies that were encouraging. For instance, some groups have published docking pipelines suitable for running in the cloud.^4,7,14−16^ Although available as open-source, these generally lack a step-by-step protocol suitable for nonexperts. Commercial solutions to docking in the cloud are also available, such as Orion (OpenEye, Santa Fe NM). This platform is currently made available at cost to the academic community.

Here, we report on a study that investigates the efficient use of AWS for docking, with a step-by-step protocol for community use. The approach is tailored to our own software but is sufficiently general that it should be easily adaptable to any docking software and perhaps even other workflows.

## Methods

Scripts are written in Python 3.8. We used the docking protocol previously outlined,^17^ and our benchmark used the successful docking parameters developed in an earlier study for the DRD4 receptor.^1^ We use the DockBlaster2.0 software for automated receptor preparation. All software for using DOCK in the cloud is freely available via https://github.com/docking-org/awsdock-scripts. This software may be forked.

### Modification of DOCK 3.8 To Be Interruptible and Restartable

Setting intentional limits on execution time can result in increased throughput for jobs in an HPC environment and possibly better prices in a cloud. Shorter jobs are more attractive to schedulers. On AWS Batch resources will be discounted if the user agrees to let others bid for them while the user is using them, with no guarantees on whether or when exactly the user’s job will be interrupted, save for a minimum 2 min warning. Batch jobs may be configured to automatically resubmit interrupted jobs to compensate for this. The configuration for restartability in the base DOCK 3.8 code is quite simple. We start a background task that checks an AWS-provided URL every 5 s, and if set, it sends a signal to the DOCK program. DOCK checks for this signal at the start of each molecule. If set, the current file index is recorded to a file named “restart” in the working directory and the program exits normally. If the restart file is present at the beginning of the program, DOCK skips to begin docking where the restart pointer points. After DOCK has exited, the output for the current run is compressed and renamed according to how many times the job has been resubmitted and then pushed to storage, e.g., OUTDOCK.gz.0, OUTDOCK.gz.1, and so forth. If the program received an interrupt signal, the restart information is also pushed to storage; otherwise, the restart file is deleted from storage. In practice, we find that typically fewer than 1% of jobs in AWS are interrupted and require a restart, and perhaps, 0.1% or less require 2 or 3 restarts. We support up to 10 restarts and only rarely see 4. Our work units are 5000 up to molecules.

### Aws-Setup Tool

The aws-setup tool can be generalized to configure all sorts of cluster-based computation environments in the cloud. Our aws-setup container image by default comes with a configuration for creating a docking environment, as well as a script to submit jobs to that environment (similar to our SLURM submission script, subdock.bash).

### License UCSF DOCK 3.8

We used UCSF DOCK 3.8.0 for all docking in this work. DOCK is free to academics at https://dock.compbio.ucsf.edu/. For for-profit outfits, DOCK 3.8 is modestly priced (write to dock_industry@googlegroups.com for more information).

### Select a Database Subset

We used ZINC-22^18^ as of September 2022 (4.5 billion lead-like molecules). To select a database subset, we should browse https://cartblanche22.docking.org/ and select Tranches >3D. Then, we should use the selection tool to select the desired region of chemical space. When we are ready, we should download the selection for db2.tgz format and select for the AWS platform.

### Obtain Sample Data for DUDEZ for Docking

We should browse https://dudez2022.docking.org/ and download either the tarball for each target or all targets together.

### Prepare Docking Parameters

We should browse https://tldr.docking.org, login, and go to the start/dockopt module and upload the target information. Then, we should click “Go” and wait about an hour to be informed that the receptor preparation job is complete and that the results are ready for download. Sample data are available at https://dudez2022.docking.org/.

### Benchmarking Calculations

We docked a database of around 50 million commercially available small molecules with a heavy atom count of 22 for benchmarking (Table 1). The actual molecules docked may be found at https://dudez2022.docking.org/. The docking model used for the benchmarking was taken exactly from our previous work on dopamine DRD4^1^ and is available for reference at https://dudez2022.docking.org/.

**Table 1:** Cost to Dock 50 Million Molecules, HAC 22, to the DRD4 Receptor, Standard Protocol (See Methods)^a^

zone	cost (esd)	variant	observation
us-east-2	$94(5)		lowest cost
us-east-1	$186(6)		200%
us-west-1	$170(5)		181%
us-west-2	$220(7)		230%
eu-north-1	$152(5)		162%
sa-east-1	$280(10)		300%
us-east-2	$127(23)	bid = 20%	worse than bid = 100
us-east-2	$115(12)	bid = 50%	worse than bid = 100
us-east-2	$103(10)	strategy = SCO	not significant
us-east-2	$103(10)	strategy = BFP	not significant

a Docking takes about 1 s per molecule on average. The variant column describes the variable, either bid percentage or job allocation strategy, which changes in addition to the zone where the job is run. The default job allocation strategy is the best fit, and the variants are spot capacity-optimized (SCO) and best fit progressive (BFP). The observation column interprets the relative cost of each job. Percentages are quoted compared to the lowest cost run, as indicated. All costs are in USD, and each is expressed with an estimated standard deviation (esd) in parentheses based on three runs.

### AWS Pricing

The current spot pricing in each zone can be viewed at https://aws.amazon.com/ec2/spot/pricing/ and compared with the full price of a reservation here https://aws.amazon.com/ec2/pricing/on-demand/. While these enable an estimate of the upper and lower bounds of a calculation, we found that the actual price varied a lot, often unpredictably (Table 2).

**Table 2:** Cost of On-Demand and Spot Instances on AWS for Two Representative Compute-Optimized Instance Types^a^

zone	C5.18× large on-demand	C5.18× large spot	C5.large on-demand	C5.large spot
us-east-1	306	117 (+44%)	8.5	3.54 (+82%)
us-east-2	306	81.2	8.5	1.95
us-west-1	382 (+25%)	111 (+36%)	10.6 (+25%)	3.08 (+60%)
us-west-2	306	116 (+43%)	8.5	3.26 (+67%)
eu-north-1	328 (+7%)	98.3 (+21%)	9.1 (+7%)	2.73 (+40%)
sa-east-1	472 (+54%)	179 (+220%)	13.1 (+54%)	4.7 (+241%)

a US cents (¢) per hour. C5.18× large has 72 cores and C5.large has 2 cores. Standard deviation estimates (esd) are in parentheses.

CPU is generally the largest cost of working in the cloud, but other charges can still contribute. These include the cost of persistent storage in S3 buckets, data transfer between various availability zones in AWS, and egress charges to download data. Uploading data to AWS is free. A detailed breakdown of these costs, with all the complexities of volume-based discounts, zone-based costs, and differential pricing for transfer among zones, is too complex to describe fully here and is covered in detail on the AWS website, but some generalizations are possible. In our experience, data transfer between zones can be up to 4% of the CPU cost and can be nearly zero if work is confined to a single zone. Storage costs in S3 were 1–2% of the CPU cost but can persist over time with additional charges if the data are not deleted after use. Cloudwatch costs, the cost of monitoring jobs with real-time information, cost us 0.5% of the CPU cost, but only because we were watching so closely as we wrote this paper. These charges could be almost zero if monitoring is limited. Obviously, these ratios depend on usage details. Data transfer between us-east-1 and us-east-2 is 1 cent per GB. Between us-east and us-west, the cost is 2 cents per GB. Transferring data between Europe and North America costs the same, just 2 cents per GB, but to and from South America costs 14 cents per GB. These ballpark figures are subject to volume and other discounts. S3 storage is available in tiers. We recommend the usage of “intelligent tiering” which attempts to automatically optimize storage costs and performance and costs 2.3 cents per GB per month. Long-term storage “Glacier” can cost as little as 0.36 cents per GB per month. There are many options and an entire industry focused around optimizing AWS costs.

### Carbon Footprint of Calculations

Docking calculations have an environmental impact. The actual carbon footprint of any particular calculation is challenging to calculate with precision because of the many variables involved, including not only the power drawn by the CPU and room cooling but also the cost of hardware manufacture, delivery, storage, and disposal. Still, Amazon provides estimates of these values in Amazon’s own Customer Carbon Footprint tool. This tool tells us that in every month that we did not exceed $10,000 in CPU costs, our carbon footprint was entirely offset by AWS renewable energy purchases. In those months where we spent $15,000, for instance, our estimated net CO_2_ footprint was 4.8 MTCO2e (million tons of CO_2_ equivalent). In this same month, 9.9 MTCO2e were saved by AWS renewable energy purchases and 3.5 MTCO2e were estimated to be saved by using AWS instead of on-premise computing, which may be less efficiently used than AWS hardware.

## Results

In principle, a cloud system like AWS provides almost unlimited resources for virtual screens that would otherwise be compute-bound as we now find with ultralarge library docking. In practice, these calculations can be unaffordable versus in-house screens, unless optimized for cost and throughput. We investigated three variables that impact the speed and cost of a large library calculation: the availability zone where the calculation is run, the resource allocation strategy, and the bid percentage, i.e., how much we are prepared to pay to avoid temporarily surrendering cores to others willing to pay more for them. We examined the impact of each of these variables on the cost of a screen of a set of 50 million midsized (HAC 22), lead-like molecules against the DRD4 receptor. The results of the experiments are summarized below (Table 1).

We wondered whether docking in different availability zone regions would help with throughput, by adding more CPUs, and what the impact on total cost would be. AWS currently supports over 17 availability zone regions worldwide with more in development, many with multiple data centers. To consider the best one for docking, the prospective user should consider both the differential cost of the CPUs in different zones and the cost of data transport between regions. We began by considering the relative on-demand and spot prices of two representative instance types in six representative zones (Table 2). On-demand means a machine is reserved exclusively for our use until we surrender it, while spot means we are willing to be interrupted at any time, with a 2 min warning. To support this use, DOCK 3.8 was modified to be interruptible and restartable to be compatible with the AWS conventions for spot instances. This data suggests that us-east-2 should be the cheapest zone to run spot calculations in and that the next cheapest should be eu-north-1, perhaps somewhere between 20 and 40% more expensive than us-east-2.

We performed our standard docking calculation (DRD4 receptor, HAC 22, 50 million molecules) in six zones to test price sensitivity. Each experiment was run three times at randomly selected times over a period of 2 weeks to assess variability as the jobs compete with the background of all other jobs. Consistent with the spot pricing we were quoted, us-east-2 was clearly the cheapest to run docking calculations. Eu-north-1 came in second, costing a 50% premium, us-west-1 at a 70% premium, and us-east-1 at twice the cost of the baseline us-east-2 pricing. The most expensive zones, as predicted by the spot prices we selected, were sa-east-1 at 300% and us-west-2 at 230% of us-east-2. While the spot price estimates captured much of this, the estimates derived from quoted prices were optimistic in our hands, making us-east-2 even more competitive than we had predicted.

In dollar terms, the numbers are sobering. Thus, it costs 94 dollars to dock our 50 million molecule test database in our test conditions, implying a cost of $8460 to dock 4.5 billion. eu-north-1 and us-west-1 are tied for second place among the zones we tested with a cost of $160–$170, thus about 70 to 80% more than the cheapest us-east-2. us-east-1 comes in fourth place and costs about twice as much as us-east-2, even though us-east-1 is where ZINC-22 is hosted in S3 and where our S3 bucket of results resides. Us-west-2 comes in fifth at $220, thus about 2.3× the cost of running in us-east-2. Finally, sa-east-1 comes with $280 or about 3× the cost of us-east-2.

Data transport costs, while generally minor, could be surprising for some zones. Notably, transporting results back from sa-east-1 to us-east-1 where our S3 bucket lives cost about 15c per GB, for 50 million molecules about $20. Not a lot, $20, but at a 4.5 billion scale that would be $1800 in data transfer costs alone. Other zones we tested are considerably cheaper to transport back from, about 1/10th as much, or $180 for a full screen. This cost could be reduced by merging results locally within the remote zone and only transporting the final top 100,000 scoring molecules of each 50 million set back to us-east-1.

Spot instances use spare compute capacity that is available for less than the price of a fixed reservation. A lower (“spot”) price is available, with the proviso that we may have to give up the CPU on short notice if another user is prepared to pay more. We figured that if we capped our bid at less than the recommended 100% of the CPU cost, we might save some money, but perhaps at the expense of being evicted more frequently, possibly incurring additional costs. We tested this trade-off by docking using the following maximum bid levels: 100% (default), 50%, and 20%. Because of the random element of contention with other jobs in real time, we performed each run five times to obtain mean and standard deviation value estimates (esd). Surprisingly to us, we found (Table 1) that bidding less than 100% was almost always more expensive than bidding 100%. We saw a savings of 20% in a single run at 50% bid, but for most runs, lower maximum bids, whether of 20%, 50%, or even 90%, were generally 20–30% more expensive. This appears to be due to more frequent evictions, which add to the cost. As a result, bidding 100% (the default) was the best choice, on average, and is used in our protocol.

Another choice we explored is the resource allocation strategy. We wondered how it affected the price and the speed of scheduling jobs. The three choices are BEST_FIT, BEST_FIT_PROGRESSIVE, and SPOT_CAPACITY_OPTIMIZED. These different options affect how machines are selected for running jobs and use different strategies that explore the trade-off between cost and throughput in response to current usage on the system.^19^ Our experimental results (Table 1) failed to distinguish these three methods despite repeated attempts. We decided to use BEST_FIT_PROGRESSIVE as our default allocation strategy.

We were curious to compare the cost and speed of running in AWS with the use of our own departmental cluster (Table 3). By assuming some values, we could estimate the costs of various idealized configurations. We considered three configurations as follows: a small cluster (128 cores in a single machine) corresponding to a small lab, a medium-sized cluster, 10 units of 128 cores each, similar to our own lab cluster, and a bigger cluster, 40 units of 128 cores, similar in magnitude to our shared cluster at UCSF. We assumed that 128 cores cost $15,000, a reasonable estimate given in recent experience and current market conditions. It is easy to redo the calculations with your own costs. We assumed that the all-in cost to keep one server running is 2/3 of its cost per year; thus, we budget $10,000 annually for electricity, cooling, rack space, and system administration time. Within the university, obtaining such values can be challenging. In each configuration, we buy 1 PB of disk storage to hold 4.5 billion molecules of ZINC-22 in db2.tgz format with some additional storage for docking results. We assumed three scenarios using AWS: (a) one LSD job per year, (b) 10 LSD runs per year, and (c) 38 LSD runs per year, corresponding to the largest cluster we considered.

**Table 3:** Comparison of the Cost of Running Docking Jobs in AWS vs on an In-House Cluster^a^

item	local 128 cores	local 1280 cores	local 5120 cores	AWS
initial cost	$65k	$200k	$650k	$0
annual cost	$10k	$100k	$400k	$0
cost/dockjob	$0	$0	$0	$25k
dockjobs/yr	0.9	9	36	28^b^
days/dockjob	407	41	10.5	13

config.	year 1	year 2	years 1–5	#jobs/5 yrs	cost/dockjob
local 128	$75k	$10k	$115k	4.5	$25.6k
local 1280	$300k	$100k	$700k	45	$15.6k
local 5120	$1050k	$400k	$2650k	180	$15.6k
AWS 1/yr	$25k	$25k	$125k	5	$25k
AWS 10/yr	$250k	$250k	$1250k	50	$25k
AWS 28/yr	$700k	$700k	$3500k	140	$25k

a A dockjob considered here screens 4.5 billion molecules, the current size of ZINC-22. We assume docking averages 1 s per molecule, which is typical but varies by target and parameter choices.

b The number of AWS jobs per year is effectively unlimited. The choice of 28 jobs per year is an illustrative example for comparison with on-premise hardware.

We looked at the dollar cost per docking job, the wall clock time to execute a single large-scale screen, and the number of docking jobs that can be executed per year.

The time to execute a large-scale docking screen (4.5 billion molecules. 1 s/mol) varied considerably between our various models. On a single 128-core machine, it takes more than a year to run. On 10 times as many (1280 cores), it takes 41 days, almost 6 weeks. On 4 times as many again, 5120 cores, a full screen takes under 11 days. Compare these figures with AWS, where an average of 4000 cores gets a 4.5 billion molecule docking job run in 13 days.

The cost to execute a large-scale docking screen also varied. Thus, a single machine with 128 cores costs $25.5k for a single screen. This is high partly because of the upfront cost of the disk required to hold ZINC-22. Increasing this cluster 10-fold, so 1280 cores, reduced the cost to only $15.6k per large-scale screen, albeit with a higher upfront investment of $300k in year 1 (all in, including space and expert/sysadmin). Quadrupling this cluster to 5120 cores does not decrease the cost per job. On AWS, we are able to access about 4000 cores in varying price categories, ranging from about $10,000 to over $30,000. We have set a price of $25k per 4.5 billion molecules, which allows running in several higher-priced zones. Running in the most economical zones can reduce this cost by half or more but at correspondingly slower throughput. In short, investing in a sufficiently larger in-house cluster, with its associated support costs, may often be more cost-efficient than AWS, but it does come with a substantial investment, while AWS may be spun up or down at will, on a job-by-job basis, without affecting per-job costs.

Equipped with the results of these experimental tests to optimize our protocol, we compiled a step-by-step procedure in five stages to run jobs efficiently in AWS, as follows (Table 4).

**Table 4:** Steps to Run LSD in the Cloud

stage of LSD	Supporting Information	online at https://wiki.docking.org/index.php/...
set up account	S1	AWS/Set_up_account
upload files for docking	S2	AWS/Upload_files _for_docking
submit docking job	S3	AWS/Submit_docking_job
merge and download results	S4	AWS/Merge_and_download_results
cleanup	S5	AWS/Cleanup

### Stage 1. Assessment of the Cost and Difficulty of Setting Up a Cloud Account for Running Large-Scale Docking

Setting up an AWS account for the first time and preparing all the settings manually is a complex task with many steps and numerous pitfalls. We investigated ways to simplify this process and solved it by writing a script and a protocol that reduces the hundreds of steps needed to just seven. As a result, it now takes only 10 min to set up an AWS account that is fully ready to run LSD. The step-by-step procedure is given in Supporting Information S1 and reference (20). Setting up an account requires setting up a payment method but incurs only minor charges. This step includes creating an S3 “bucket” to contain the project files and creating a docker image to run the calculations.

### Stage 2. Assessment of the Cost and Difficulty of Preparing a Job for Large-Scale Docking

Preparing to run a large-scale docking calculation has three steps. The docking model, a description of the protein binding site used by DOCK to score library molecules, must be prepared and parameterized, the part of chemical space to be screened must be selected, and these two items must be uploaded to AWS. Docking model preparation can be done in three ways: (A) by licensing DOCK 3.8 (via dock.compbio.ucsf.edu) and running it locally and (B) automatically using our website TLDR (tldr.docking.org, free registration required), using the dockopt module. This approach currently requires that the user has known ligands and decoys available. (C) Use prepared files for any of the 42 DUDEZ targets available from our website, dudez2022.docking.org. The chemical space to be screened may be selected and downloaded using the 3D Tranche Browser in ZINC-22 (cartblanche22.docking.org). All the relevant files should be uploaded to an S3 bucket on the user’s AWS account using the aws command line tool (awscli). These steps are described in Supporting Information S2.

### Stage 3. Assessment of the Cost and Difficulty of Running a Large-Scale Docking Screen

Once all of the required inputs are ready, launching a docking calculation is relatively simple. We have developed some recommended best practices that help to keep the calculation organized and allow late-stage decisions about how to organize the results. A script and a protocol describing this step are given in Supporting Information S3. This step is where almost all of the cost of LSD is incurred. Our protocol recommends starting with a fraction of the database to dock to test that the calculation is producing desired results. In this way, $50 can be spent early to produce final results, which can be checked carefully before more funds are committed. The most basic is to check that the molecules are being docked in the correct site, are fitting and scoring as might be expected, and that the user would consider purchasing at least some of the top-scoring molecules. A more complete description may be found in our docking protocol.^17^ If the results are not satisfactory, the user should reconsider the docking model, perhaps by seeking advice from a local expert. When a satisfactory pilot screen has been run and checked, a full screen, costing perhaps tens of thousands of dollars may be launched, with the confidence that the calculation is producing desirable top-scoring molecules.

### Stage 4. Assessment of the Cost and Difficulty of Merging the Final Results and Preparing the Result for Manual Review and Prioritization for Purchase

When docking is complete, the user may combine the docking results to yield one or a few lists of top-scoring ligands for clustering and review (see Supporting Information S4).

### Stage 5. Assessment of the Difficulty of Cleaning Up the AWS Account When the Calculation Is Complete

When the compounds have been ordered, the user may wish to reduce the disk space usage in AWS to reduce or avoid monthly charges. We propose several strategies, each with advantages and disadvantages. See Supporting Information S5.

A step-by-step protocol emerging from these studies is summarized in Table 4. While we exemplify the use of AWS with DOCK 3.8, we believe that many of the steps described may be applied to other docking software and perhaps even software other than docking. Of course, such programs should be interruptible and restartable to take advantage of spot pricing. Such adaptations of other docking software should not be difficult for a software developer.

We have tested this protocol using the DUDEZ benchmark.^21^ Ready-to-use inputs together with sample outputs are publicly accessible on our website, dudez2022.docking.org. Using these data, the user may test whether our protocol has been followed correctly.

## Discussion

Three themes emerge from this work. First, large-scale docking at a multibillion molecule scale is now possible in the AWS cloud with UCSF DOCK 3.8—we suspect that these procedures could be readily adapted to many docking programs. The calculation requires no commercial licenses for academics. Billions of commercially available molecules can be screened, typically for around $25,000 or less. No upfront investment in infrastructure and personnel to maintain it is required. Second, we have explored some of the cloud parameter choices for their effect on the cost and speed of jobs. The best parameters we could find have been incorporated into our standard pipeline and best practices. Third, new software tools and a detailed protocol are freely available to simplify all aspects of docking in the cloud, from configuring a cloud account, to prosecuting the screen, to merging and downloading the final results. We take up each of these points in turn.

Docking multiple billions of molecules is no longer limited to experts with large computer clusters and expertise in cloud computing. Using the approach described here, any investigator can run LSD screens without assembling an expensive computer lab, without engaging costly consultants in cloud computing, and without entangling alliances with commercial software vendors. Our approach does not use any graphical user interfaces, does not require any licenses other than to DOCK itself, and does not require licensing any third-party software not already included in DOCK. We focused on using tools that are native to AWS, adapting UCSF DOCK 3.8 to play within the constraints of that ecosystem. DOCK is now interruptible and restartable allowing the use of cheaper instances with spot pricing. Our protocol is intrinsically conservative, allowing a calculation to be tested at a small scale at each step to avoid wasting money. There is scope for both improvisation and adaptation in our protocol because our approach is simple, modular, and open.

There are numerous parameter choices to be made in the cloud that govern how the calculation is scheduled, where the data are stored, and how much the user is willing to pay to run jobs. We have examined three sets of choices empirically and have repeated the calculations to explore the variability of the cost of using the shared, interruptible resource that the cloud is. By far, the most important consideration is the availability zone, where we found variations of 300% in cost among zones. The best choices have been incorporated as defaults in our scripts and protocols. Surprisingly, writing data back to us-east-2 from eu-north-1 was both fast and economical, and storing the data in a single bucket in a single zone simplified the merging of the results at the end.

Our calculation of costs and times comparing different configurations of lab clusters with the cloud shows there are interesting trade-offs to be had. It seems that buying and maintaining a home cluster of sufficient size (here, 1280 cores) is cheaper than renting (AWS), at least amortized over a 5 year period, if the machines are kept saturated (over the first year, it is far more expensive, unamortized). An underutilized on-premise cluster could easily become more expensive than AWS per job if not used efficiently. In short, investing in a sufficiently larger in-house cluster, with its associated support costs, may often be more cost-efficient than AWS, but it does come with a substantial investment, while AWS may be spun up or down at will, on a job-by-job basis, without affecting per-job costs. For occasional or uneven use, AWS provides tremendous flexibility to run jobs with no upfront cost, no long-term commitment, and reasonable cost.

Software tools and a protocol that use them have been developed and are freely available and ready to use. The software (other than DOCK itself) is available as open source on GitHub (see Methods). Our approach is not a black box but a series of simplified steps, the results of which may be inspected and tested at each stage and corrected if necessary. Parts of the protocol are highly tailored to AWS, while others are general and could be used for other cloud providers. Although the scripts all use DOCK 3.8 as the docking engine, they could be easily adapted to other docking software and perhaps even other approaches. The software is written to be used either interactively or noninteractively, allowing for a range of experiences by the target user. As much as possible, the protocol is outlined in both general, schematic terms as well as detailed step-by-step instructions. In this way, we hope that the protocol will be useful for both beginners as well as for adaptation and improvisation by experts. We have provided test data for 42 DUDEZ systems with which our protocol can be used. We also provide some sample output data, allowing users to test that they have followed our protocol correctly before embarking on their own research.

Our approach has limitations. Careful controls, when possible, including the DUDEZ benchmark, are an important way to assess a docking model and its likelihood for prospective screening success. Our automatic preparation of docking parameters currently requires actives and decoys to help optimize it, and even with these controls, it will not work well on every target. Some docking calculations can take substantially longer than others, based on the size of the binding site, degree of sampling, and size of the database screened, among other factors. This can result in widely varying costs of a large-scale docking calculation, including unexpectedly high costs. In this work, we have estimated costs based on a throughput of 1 s per library molecule, which is typical for DOCK3.8 but can certainly vary. We recommend small-scale calculations to estimate the total cost before attempting a run at scale. Suboptimal parameter choices can easily double the length (and cost!) of the calculation. Our protocol has been tested in six availability zones and should work in any AWS zone, but we have not been able to test it everywhere yet.

Notwithstanding these caveats, a new protocol for docking at scale in the cloud is now available. The step-by-step, detailed protocol requires no specialist expertise and in particular no prior experience with AWS. Their adoption should lower barriers to entry to large library docking in the community, bringing the large chemical space they represent to ever more interesting biological targets.

## Conclusions

Running large-scale molecular docking screens in the AWS cloud is now pragmatic and does not require any commercial software. The procedures to do so are outlined here in step-by-step detail. A typical screen of 4.5 billion molecules using DOCK 3.8 should cost around $25,000, a cost which may be cheaper than establishing and supporting an on-premise computer cluster.

## Software Availability

All software for using DOCK in the cloud is freely available via https://github.com/docking-org/awsdock-scripts.

## Supporting Information Available

Summary of steps for docking (PDF)
AWS: Set up an account (PDF)
AWS: Upload files for docking (PDF)
AWS: Submit docking job (PDF)
AWS: Merge and download results (PDF)
AWS: Cleanup (PDF)
