# Supplementary material for: Large-Scale Docking in the Cloud
Source: J Chem Inf Model. 2023 Apr 18;63(9):2735–41. doi: 10.1021/acs.jcim.3c00031 (PMC10170500; doi:10.1021/acs.jcim.3c00031)
Supplement: Supplementary file 1 — ci3c00031_si_001.pdf [file ci3c00031_si_001.pdf]

Supporting Information for

Large Scale Docking in the Cloud

Benjamin I. Tingle and John J Irwin*

University of California San Francisco, Department of Pharmaceutical Chemistry, 1700 4th St,

MC 2550, San Francisco CA 94158-2330

Corresponding author jji@cgl.ucsf.edu

Table of Contents

Each file is a separate PDF document

S1. AWS:Set up account

S2. AWS:Upload files for docking

S3. AWS: Submit docking job

S4. AWS:Merge and download results

S5. AWS: Cleanup
