# Supplementary material for: Large-Scale Docking in the Cloud
Source: J Chem Inf Model. 2023 Apr 18;63(9):2735–41. doi: 10.1021/acs.jcim.3c00031 (PMC10170500; doi:10.1021/acs.jcim.3c00031)
Supplement: Supplementary file 2 — ci3c00031_si_002.pdf [file ci3c00031_si_002.pdf]

AWS:Set up account

- Tutorial 1 AWS:Set up account THIS TUTORIAL
- Tutorial 2: AWS:Upload files for docking
- Tutorial 3: AWS:Submit docking job
- Tutorial 4: AWS:Merge and download results
- Tutorial 5: AWS:Cleanup

Contents

Installation

Container Environment

Quickstart - Creating your first AWS docking environment

- Setup
  - Credentials & Region
  - S3 Bucket
  - First time setup
- Environment Creation
  - Bid Percentage
- Advanced Usage

Installation

Docker is required to run the aws-setup scripts. https://www.docker.com/get-started/. You can install docker desktop to your personal machine, or log on to a machine where docker is already installed.

An Amazon AWS account is also required, with payment attached. https://aws.amazon.com/premiumsupport/knowledge-center/create-and-activate-aws-account/

On a linux/mac/windows computer with docker or docker desktop installed, run the following commands in a terminal:

```
docker pull dockingorg/aws-setup
docker run -v /var/run/docker.sock:/var/run/docker.sock --rm -it dockingorg/aws-setup
```

Explanation of arguments:

- `-v /var/run/docker.sock:/var/run/docker.sock` Allows the container to use your system's Docker
- `--rm` Cleans up the container once you've exited
- `-it` Runs the container interactively

It may be necessary to give the container additional privileges. When you enter the image, test this with the following command:

```
root@f54f423d64b1:/home/awsuser# docker ps
```

If you get a permission denied error, exit the container and run again with the --privileged option enabled:

```
docker run --privileged --rm -v /var/run/docker.sock:/var/run/docker.sock -it dockingorg/aws-setup
```

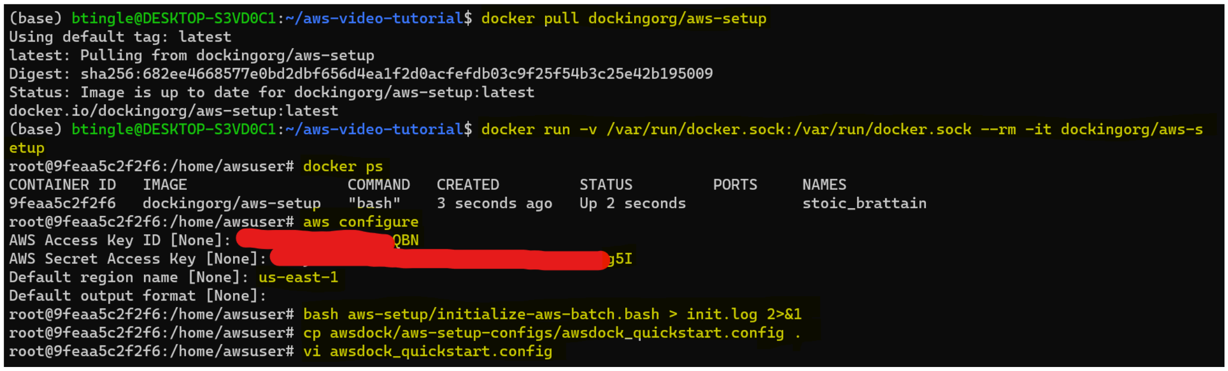

```
(base) btingle@DESKTOP-S3VD0C1:~/aws-video-tutorial$ docker pull dockingorg/aws-setup
Using default tag: latest
latest: Pulling from dockingorg/aws-setup
Digest: sha256:682ee4668577e0bd2dbf656d4ea1f2d0acfefdb03c9f25f54b3c25e42b195009
Status: Image is up to date for dockingorg/aws-setup:latest
docker.io/dockingorg/aws-setup:latest
(base) btingle@DESKTOP-S3VD0C1:~/aws-video-tutorial$ docker run -v /var/run/docker.sock:/var/run/docker.sock --rm -it dockingorg/aws-s
etup
root@9feaa5c2f2f6:/home/awsuser# docker ps
CONTAINER ID   IMAGE                  COMMAND   CREATED         STATUS         PORTS     NAMES
9feaa5c2f2f6   dockingorg/aws-setup   "bash"    3 seconds ago   Up 2 seconds             stoic_brattain
root@9feaa5c2f2f6:/home/awsuser# aws configure
AWS Access Key ID [None]: [redacted]
AWS Secret Access Key [None]: [redacted]
Default region name [None]: us-east-1
Default output format [None]:
root@9feaa5c2f2f6:/home/awsuser# bash aws-setup/initialize-aws-batch.bash > init.log 2>&1
root@9feaa5c2f2f6:/home/awsuser# cp awsdock/aws-setup-configs/awsdock_quickstart.config .
root@9feaa5c2f2f6:/home/awsuser# vi awsdock_quickstart.config
```

Container Environment

The container uses the ubuntu distribution. Some utilities such as curl and vi are installed so you can download files and edit them. You can also install whatever software you like using "apt install", e.g "apt install git".

If you have files you'd like to access from the container, you can link them in using the docker "-v" option. By default we link the docker socket using this option ("-v /var/run/docker.sock:/var/run/docker.sock"), but you can link any number of directories or files in this manner. For example, if you would like the contents of the "/tmp" directory on your local machine to be available under "/temp" in the docker image, you would add the following option to your "docker run" command: "-v /tmp:/temp", for a final command of:

```
docker run -v /tmp:/temp -v /var/run/docker.sock:/var/run/docker.sock --rm -it dockingorg/aws-setup:latest
```

If you're an advanced user and you'd like to create your own version of the aws-setup image with certain software preinstalled, you can request us for access to the aws-setup repository, which contains the scripts and Dockerfile we use to set up the docker image. You can also build your own image using our aws-setup image as a base.

Quickstart - Creating your first AWS docking environment

Setup

Credentials & Region

When you enter the docker image, you will be in /home/awsuser. There should be two directories in front of you, aws-setup and awsdock. We start off by going into the aws-setup directory and configuring our AWS credentials. (This needs to be done every time you log in to the container)

```
root@f54f423d64b1:/home/awsuser# cd aws-setup
root@f54f423d64b1:/home/awsuser# aws configure
```

You'll now be prompted to enter your AWS access key ID & AWS secret access key. If you already know what these are you can enter them and move on. If you don't know what your AWS secret key and access key are, follow this tutorial: https://aws.amazon.com/blogs/security/wheres-my-secret-access-key/. Make sure to save your keys somewhere safe that you will remember!!

Next, you'll be prompted on which AWS region you would like to use. If this is your first environment, set the region to us-east-1. Our lab's molecule data S3 bucket (zinc3d) is also located in this region, so this is the most economical region to run docking jobs in, due to the cost of moving data between AWS regions. (see diagram)

More info on regions & region codes here: https://docs.aws.amazon.com/AWSEC2/latest/UserGuide/using-regions-availability-zones.html

The last prompt sets the preferred output format- feel free to leave this blank, or set it to "json".

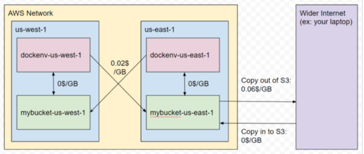

Diagram showing the cost of transferring S3 data between regions and across to the internet

S3 Bucket

An S3 bucket is a virtual hard drive that your AWS resources can access from anywhere. You will need to create one on your account prior to creating your AWS environment. Follow the amazon tutorial on how to do this: https://docs.aws.amazon.com/AmazonS3/latest/userguide/create-bucket-overview.html

The quickstart guide will show you how to create an AWS environment in us-east-1, so it is best to create your S3 bucket in this region.

It is best to have a dedicated S3 bucket for each region you create an environment for, due to the cost of inter-region data transfer.

First time setup

If it is your first time setting up an environment on your AWS account, you will need to run initialize-aws-batch.bash. This script only needs to be run once per account.

```
root@f54f423d64b1:/home/awsuser/aws-setup# bash initialize-aws-batch.bash
```

You should see this script spit out a bunch of JSON text. If you accidentally run this script when it has already been run before, you may see a bunch of errors along the lines of: "Service role name <blank> has been taken in this account". Don't worry about these, they don't mean anything.

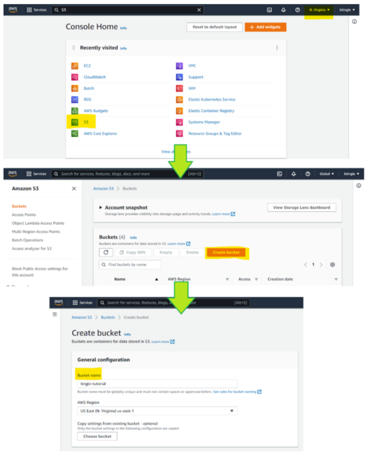
Diagram explaining how to create an S3 bucket. Note the region- N.Virginia aka us-east-1. This is the optimal region for running docking.

Environment Creation

```
root@f54f423d64b1:/home/awsuser/aws-setup# bash create-aws-batch-env.bash /home/awsuser/awsdock/aws-setup-configs/awsdock_quickstart.config
```

The quickstart configuration will name your environment "dockenv-us-east-1". This name serves as the unique identifier for this environment, you'll refer to it later when submitting jobs. If you try to create an environment that already exists with the same name, the script will update the existing environment instead of creating a new one.

If you would like to set up an environment with a different name or based in a region other than us-east-1, you can use aws-setup-configs/awsdock.config instead.

Attach the bucket you created to the environment. Don't qualify this with the s3:// path, just the plain name.

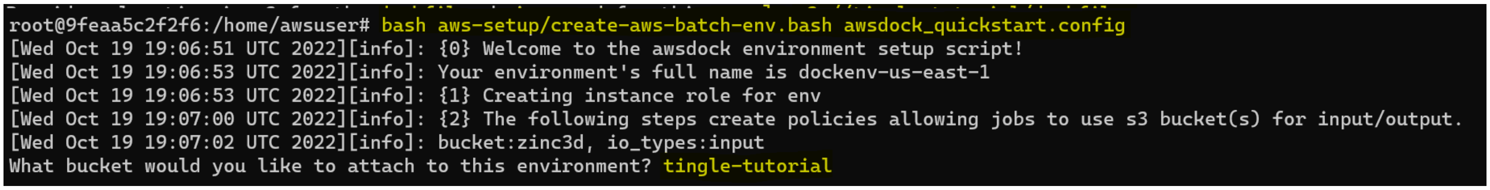

Set MAX_CPUS for your environment to desired value. This parameter refers to the maximum number of jobs that can be run in parallel. You should set this at or below the suggested value- this value is derived from the AWS imposed resource limit. You can learn more about resource limits and how to increase them at this page: Docking_Submission_On_AWS#Resource_Limits

Set BID_PERCENTAGE for your environment to desired value. See section below for more explanation of this parameter, it can potentially save you money. If you're not sure, keep the default.

```
latest: digest: sha256:9d019d18ab7670b52b86f37869ce72f6e9e0ccbdcfdbb636f100039360f819b7 size: 2421
[Wed Oct 19 20:31:38 UTC 2022][info]: IMAGE NAME: dockaws-dockenv-us-east-1
[Wed Oct 19 20:31:38 UTC 2022][info]: {4} Finalizing AWS batch components
How many CPUS would you like to allocate to this environment at maximum? [suggested: 640]: 640
What is your bid percentage threshold for spot instances? Consult the docs for more info on this parameter. [default: 100]: 100
[Wed Oct 19 20:31:47 UTC 2022][warning]: launch template already exists
```

Bid Percentage

In order to save money, our AWS batch environment uses the "spot" allocation strategy, which allows us to bid on compute resources at a discount.

The BID_PERCENTAGE parameter indicates what % of the on-demand price our environment is willing to pay for compute resources. At 50%, the environment will wait for at least a 50% discount of the on-demand price to be available before purchasing resources. At 100%, the environment will pay lower prices when they're available, but failing that will pay the full on-demand price. This is the best option for those that want to save money but also don't want to waste time.

Advanced Usage

For advanced usage of the aws-setup tool, see here: AWS DOCK Environment Setup Advanced Usage

Retrieved from "http://wiki.docking.org/index.php?title=AWS:Set_up_account&oldid=14931"

This page was last edited on 2022-10-19, at 13:38:54.
