# Supplementary material for: Large-Scale Docking in the Cloud
Source: J Chem Inf Model. 2023 Apr 18;63(9):2735–41. doi: 10.1021/acs.jcim.3c00031 (PMC10170500; doi:10.1021/acs.jcim.3c00031)
Supplement: Supplementary file 3 — ci3c00031_si_003.pdf [file ci3c00031_si_003.pdf]

AWS:Upload files for docking

- Tutorial 1: AWS:Set up account
- Tutorial 2: AWS:Upload files for docking THIS TUTORIAL
- Tutorial 3: AWS:Submit docking job
- Tutorial 4: AWS:Merge and download results
- Tutorial 5: AWS:Cleanup

Contents

Upload files for docking
How to copy data to Amazon S3
Approach 1: Use the browser console
Approach 2: AWS CLI

Upload files for docking

- You've followed the tutorial for setting up the environment: AWS DOCK Environment Setup
- Following from the previous requirement, you have the aws-setup docker image installed on your machine (we will continue using it in this tutorial)
- Likewise, you will need to know your AWS access key, secret key, etc.

How to copy data to Amazon S3

In the previous tutorial, you created an S3 bucket to set up your environment. S3 buckets act like virtual hard drives, with familiar operations like cp, ls, rm, and mv for storing & manipulating data.

In an AWS docking environment, S3 buckets are responsible for storing dockfiles, input, and output for docking runs. Typically, docking input is sourced from our lab's zinc3d bucket, which houses an enormous amount of db2 data for large docking screens, while dockfiles, output, and other configuration are saved to an environment-specific bucket. For custom screens, like DUDE-Z, input can be uploaded to & sourced from the environment-specific bucket instead.

You can take one of two approaches to get your data into an S3 bucket; the first is less complicated and more intuitive, the second is more complicated but opens more avenues for automation.

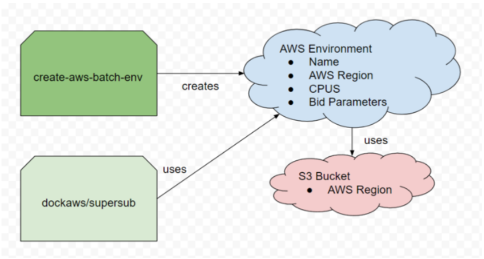

Approach 1: Use the browser console

AWS allows uploading files to S3 directly from the browser. Navigate to the S3 console (https://s3.console.aws.amazon.com/s3), where there should be a list of all buckets that your account owns.

Click on the bucket you'd like to upload to, and navigate to the folder within that bucket you want to upload to. You can upload folders and files as you wish through the interface.

You can click "Copy S3 Uri" in the top right of the interface to copy the full path of the directory or file you are viewing.

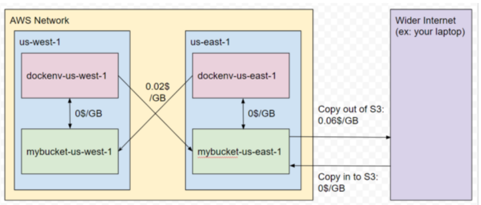

Diagram showing the cost of moving data between intra AWS regions, as well as AWS and the wider internet

Approach 2: AWS CLI

The AWS CLI provides an interface to perform any conceivable operation on AWS resources, including S3 buckets.

The CLI is available on the command line within the aws-setup image, but you may find it more convenient to install the command-line CLI locally to your machine, such that all your files etc are visible. Here is the amazon tutorial on how to install the CLI: https://docs.aws.amazon.com/cli/latest/userguide/getting-started-install.html

For example, the CLI command to copy one file from your local drive into an S3 bucket looks like this:

```
aws s3 cp myfile.txt s3://mybucket/mydir/myfile.txt
```

Note that myfile.txt was saved under mydir/myfile.txt on the S3 bucket. Directories are created implicitly in S3, meaning it is not necessary to "mkdir" to create a directory, simply including the directory in the file's path is enough.

For a practical example, say you want to copy your dockfiles to S3. Here's what that looks like:

```
aws s3 cp --recursive docking_params/5HT2A/dockfiles s3://mybucket/docking_runs/5HT2A/dockfiles
```

The --recursive argument behaves similarly to "cp -r", allowing you to upload the directory's contents with one command.

If you've been using the aws-setup container to access the AWS CLI, you may be wondering how to find your dockfiles, as your system's usual files are not visible from within the container.

You can link files on your system to the container using docker's "-v" argument. For example, say all your various docking parameters are located under /home/myuser/dockingstuff on your system, and you'd like them to be visible within the aws-setup container somewhere.

```
docker run --rm -it -v /var/run/docker.sock:/var/run/docker.sock -v /home/myuser/dockingstuff:/tmp/dockingstuff dockingorg/aws-setup:latest
```

That extra "-v" argument tells docker to make all files under /home/myuser/dockingstuff on your host system visible in the container under the /tmp/dockingstuff directory. Once we've entered the container, we can verify this is true by ls-ing the /tmp/dockingstuff directory:

```
root@65aa6738db54:/home/awsuser# ls /tmp/dockingstuff
5HT2A    something_else    README.txt    docking_is_cool.smi
```

Note- if you're a Mac user, there may be some pain with permissions during this step. Your Mac will want you to provide explicit permissions for docker to access the linked directory, here's the docker tutorial on how to fix this: https://docs.docker.com/desktop/mac/#file-sharing.

do not forget about

- DOCK 3.8:Preparing_dockfiles_using_TLDR
- ZINC-22:Select_chemical_space_for_docking
- AWS:Upload_docking_files_to_aws

Retrieved from "http://wiki.docking.org/index.php?title=AWS:Upload_files_for_docking&oldid=14907"
