# Supplementary material for: Large-Scale Docking in the Cloud
Source: J Chem Inf Model. 2023 Apr 18;63(9):2735–41. doi: 10.1021/acs.jcim.3c00031 (PMC10170500; doi:10.1021/acs.jcim.3c00031)
Supplement: Supplementary file 4 — ci3c00031_si_004.pdf [file ci3c00031_si_004.pdf]

AWS:Submit docking job

- Tutorial 1: AWS:Set up account
- Tutorial 2: AWS:Upload files for docking
- Tutorial 3: AWS:Submit docking job THIS TUTORIAL
- Tutorial 4: AWS:Merge and download results
- Tutorial 5: AWS:Cleanup

Contents

Quickstart- Submitting docking jobs through AWS
- Setup
  - Upload Dockfiles
  - Upload Input List
  - Note for first-time runs
- Job Submission
  - Alternative DOCK Executable
  - Submission Configuration
  - auto aws configure
- Monitoring Jobs
- Resource Limits
  - Viewing your current resource limits
  - Requesting resource limit increases

Quickstart- Submitting docking jobs through AWS

Setup

Upload Dockfiles

Using your preferred method, upload your dockfiles to S3. Make sure to copy the S3 URI for your dockfiles folder, it will be used during submission. INDOCK configuration is expected to be located within the dockfiles folder.

Upload Input List

The input list may need some explanation. This input list is expected to be a text file containing a list of S3 paths to dockable db2.tgz files accessible by the environment you are submitting to. For example, here is a short but valid input list:

```
s3://zinc3d/zinc-22x/H17/H17P200/a/H17P200-N-xaa.db2.tgz
s3://zinc3d/zinc-22x/H17/H17P200/a/H17P200-Q-xaa.db2.tgz
s3://zinc3d/zinc-22x/H17/H17P200/a/H17P200-N-xab.db2.tgz
```

If this is your first time running docking jobs, you can use this example list to test out your environment.

If you would like to create your own list, it is possible to select molecules to DOCK through our tranches viewer, available on cartblanche: https://cartblanche22.docking.org/tranches/3d. Use the interface to select molecules based on heavy atoms, charge, and logP. Once your selection is ready, click the download button in the top right to open the download menu. Under "method", select "DOCK37 (*.db2.tgz)" and "AWS S3" then confirm the download. This file can be used as an input list.

Once your input list has been prepared, upload it to S3 using your preferred method. Make sure to copy the S3 URI for your input list file, it will be used during submission.

Note for first-time runs

Prior to submitting a large docking campaign, it is wise to prepare a smaller run first, to check that the configuration etc. are working properly. You don't want to spend oodles of money on a docking campaign that didn't produce anything due to a broken configuration.

Job Submission

This step takes place in the aws-setup container. Run aws configure on startup as per usual. If you'd like to avoid running configure on start-up every time, see the "auto aws configure" subsection at the bottom.

Run supersub.bash without any arguments

```
cd /home/awsuser/awsdock/submit
bash supersub.bash
```

You'll be greeted by a prompt to enter the full name (or identifier) of your desired environment. If you just ran through the quickstart guide your environment will be named "dockenv-us-east-1".

```
[ What is the full name ($name-$region) of the environment to submit to? ]: dockenv-us-east-1
```

Next, it will ask you to provide an S3 location to send output to. This should be an S3 URL to a folder in your environment-specific bucket; don't worry about creating the folder if it doesn't exist, it will be created automatically.

```
[ Which s3 location should output be sent to? ]: s3://mybucket/some/output/directory
```

Enter a name for your job. Whatever you want it to be, just make sure it doesn't collide with the name of any other job in your S3 output folder.

```
[ What is the name for this batch job? ]: testjob
```

Now provide the dockfiles URL and input list URL you prepared beforehand:

```
[ Provide a location in s3 for the dockfiles being used for this run ]: s3://mybucket/stuff/dockfiles
[ Provide an s3 file location for the list of files to be evaluated by this run ]: s3://mybucket/stuff/input_list.txt
```

Think over your life decisions real quick and enter y to submit the job!

```
created 1 jobs for this batch, submit? [y/N]:
```

Alternative DOCK Executable

Do you have an experimental/specially tuned dock executable you'd like to use in lieu of the default? All you need to do is upload the DOCK executable to S3, and prior to running supersub.bash, export the following:

```
# replace path here with the URI to your special executable
$ export S3_DOCKEXEC_LOCATION=s3://mybucket/dock_alternatives/special1/dock64
$ bash supersub.bash
```

Submission Configuration

The supersub.bash script can be automated through configuration files, similar to create-aws-batch-env.bash- see "configs/exconfig.config" next to supersub.bash for an example of what a complete configuration looks like.

auto aws configure

Create a file with your aws credentials like so:

```
### aws_config.txt
AWS_ACCESS_KEY_ID=<your AWS access key>
AWS_SECRET_ACCESS_KEY=<your AWS secret key>
AWS_REGION=<desired aws region code>
```

Add this file to your "docker run" command with the --env-file option, like so:

```
docker run --rm -it -v /var/run/docker.sock:/var/run/docker.sock --env-file aws_config.txt dockingorg/aws-setup
```

Monitoring Jobs

To see the status of your jobs, it is easiest to log on to the AWS Batch Console. https://console.aws.amazon.com/batch/home

Make sure to set your region in the console to the region your DOCK environment is located.

On your dashboard, you should see an overview of the status of your jobs.

- Jobs stuck in "Pending" for too long indicate that your environment has not been set up correctly and is missing a key component. Otherwise this indicates your jobs are waiting for resources.
- Jobs in "Runnable" have no outstanding issues and are ready to run, but will only run when compute resources are available. If your jobs are stuck here for a while, you may want to review your resource limits and make sure you can actually allocate the number of machines you've requested.
- Jobs in "Starting" are initializing on a real machine
- Jobs in "Running" are running on a real machine
- Jobs in "Succeeded" have finished successfully
- Jobs in "Failed" have failed

Your jobs may be stuck in the "Runnable" status for a while. Typically these jobs will go through in a couple days (and when they start to go through, they won't stop until they're all done), though there are cases where they can be stuck in this status indefinitely. The reasons for why this might be are myriad, and you're probably best off contacting me for help (ben@tingle.org). If you want to try and figure it out on your own, see this AWS help page: https://docs.aws.amazon.com/batch/latest/userguide/troubleshooting.html#job_stuck_in_runnable.

For more information on Job Statuses and what they mean, see this page: https://docs.aws.amazon.com/batch/latest/userguide/job_states.html

Resource Limits

Unfortunately, it is not possible to allocate thousands of machines for large scale docking right off the bat. Amazon imposes restrictions on your usage, especially if your account is new.

Viewing your current resource limits

Navigate to the EC2 console: console.aws.amazon.com/ec2

In the left sidebar, click on the "Limits" tab, just above the "instances" category. In the search bar, type "Spot", and look at your results.

The entry titled "All Standard (A, C, D, H, I, M, R, T, Z) Spot Insta..." shows how many CPUs you can utilize at one time. You should modify your compute environment's MAX_VCPUs configuration to match this number.

It may be disappointing to learn that you cannot maximize your LSD calculations off the bat, but the limits are in place so that you do not hurt yourself (financially) before being experienced with using AWS.

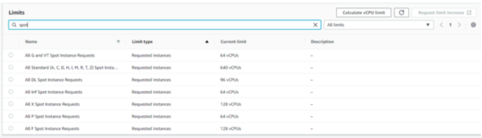

Requesting resource limit increases

In the limits menu, you can select the "All Standard (..." entry and click "Request limit increase" in the top right. In this new menu, select the region(s) you would like to increase your limit for and write a short paragraph explaining why you would like your limit to be increased. It does not hurt to set your requested limit to a reasonably high number, e.g 5000. More than likely you will not achieve this limit on your first increase, but if you shoot for the moon and miss, at least you will land among the stars, right?

Every billing cycle you can request AWS to increase your spot instance limit some more. This may take some time, but just make sure to explain your use case in the form and they will be more than happy to oblige.

Retrieved from "http://wiki.docking.org/index.php?title=AWS:Submit_docking_job&oldid=14908"

This page was last edited on 2022-10-11, at 15:11:08.
