# Supplementary material for: Large-Scale Docking in the Cloud
Source: J Chem Inf Model. 2023 Apr 18;63(9):2735–41. doi: 10.1021/acs.jcim.3c00031 (PMC10170500; doi:10.1021/acs.jcim.3c00031)
Supplement: Supplementary file 5 — ci3c00031_si_005.pdf [file ci3c00031_si_005.pdf]

AWS:Merge and download results

- Tutorial 1: AWS:Set up account
- Tutorial 2: AWS:Upload files for docking
- Tutorial 3: AWS:Submit docking job
- Tutorial 4: AWS:Merge and download results THIS TUTORIAL
- Tutorial 5: AWS:Cleanup

Contents

Prerequisites

Step 1: Create an EC2 instance

Step 2: Connect to your EC2 instance and install required software

Step 3: Configure AWS, Copy data from S3

Step 4: Run Analysis and save results

Prerequisites

github personal access token & DOCK github repository access rights (https://docs.github.com/en/authentication/keeping-your-account-and-data-secure/creating-a-personal-access-token)

You will need to provide this access token as a password when pulling the DOCK repository. You can email us at bkslab for access to the DOCK repository.

aws account & credentials (https://docs.aws.amazon.com/general/latest/gr/aws-sec-cred-types.html#access-keys-and-secret-access-keys)

Step 1: Create an EC2 instance

Go to AWS dashboard, look up EC2 in the search bar and click the link.

Navigate to "instances" tab and select "Launch Instance"

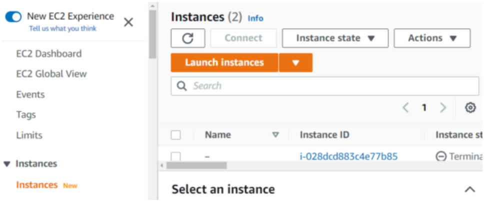

For Instance Type, find an instance appropriate for your analysis workload. You can't go wrong with more vCPUs and Memory, so a t2.xlarge or t2.2xlarge will work just fine for just about any workload.

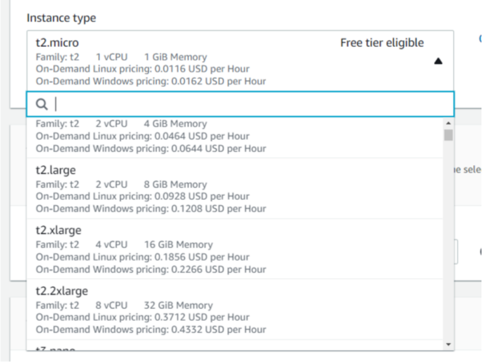

Word to the wise: if you're going to allocate an expensive instance, MAKE SURE NOT TO FORGET ABOUT IT! You don't want a surprise 1000$ bill at the end of the month for an instance you barely used.

Next, create a new key pair for your instance. Use RSA encryption and give it a descriptive name, making sure to save the .pem or .ppk file somewhere you will remember it. This key can be re-used for any additional instances you create in the future.

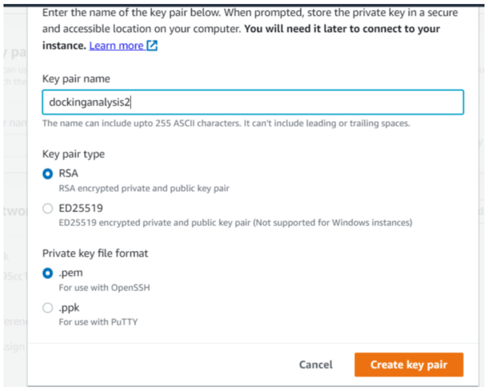

Finally, configure the storage available to your instance. Depending on how many poses your docking run produced, you may want just a few GB, or a few TB.

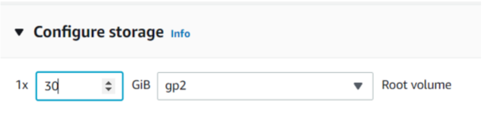

Now launch your instance, this will bring you to a page showing your instance's ID. Click on that ID to view the details of your instance.

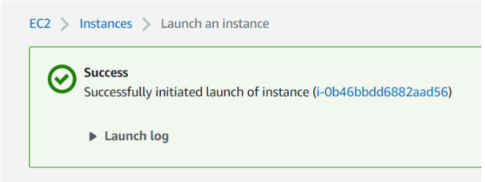

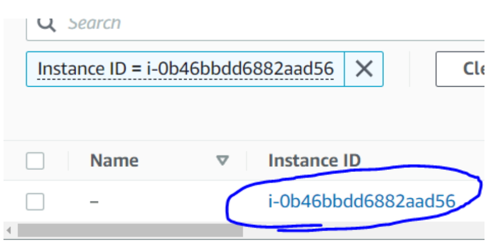

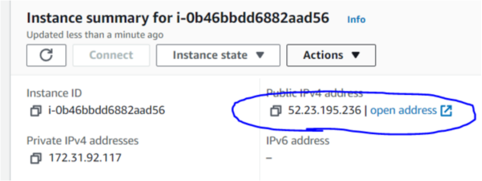

Step 2: Connect to your EC2 instance and install required software

In your instance's details page, find it's public ipv4 address.

Using your preferred ssh client, connect to your instance. The default username on ec2 instances is ec2-user, meaning your ssh command should look as follows:

```
ssh -i /path/to/my/key.pem ec2-user@54.221.XXX.XXX
```

Once logged in to your instance, you will need to install all dependencies:

- git
- DOCK
- python3.8

git:

```
sudo yum install git
```

DOCK:

```
$ git clone https://github.com/docking-org/DOCK.git
Cloning into 'DOCK'...
Username for 'https://github.com': myuser
Password for 'https://myuser@github.com': <paste your github access token here>
```

python3.8:

```
sudo amazon-linux-extras enable python3.8
sudo yum install python3.8
```

Step 3: Configure AWS, Copy data from S3

First, configure your aws credentials.

```
~$ aws configure
AWS Access Key ID [None]: MY_ACCESS_KEY
AWS Secret Access Key [None]: MY_SECRET_ACCESS_KEY
Default region name [None]: us-west-1
Default output format [None]:
```

Enter your access keys, and the region you ran docking in. Leave output format blank (or don't, it really doesn't matter).

Find the location where DOCK wrote output to in your S3 bucket, e.g

```
~$ aws s3 ls s3://btingletestbucket/docking_runs/5HT2A/H17/
                           PRE aa/
                           PRE ab/
~$ aws s3 ls s3://btingletestbucket/docking_runs/5HT2A/H17/aa/
                           PRE 1/
                           PRE 2/
                           ...
                           PRE 10000/
~$ aws s3 ls s3://btingletestbucket/docking_runs/5HT2A/H17/aa/1/
2021-03-16 22:50:57    2111052 OUTDOCK.0
2021-03-16 22:50:58    4705515 test.mol2.gz.0
```

Copy all the results you want to analyze to your instance. You don't need OUTDOCK to do analysis any more, so you can save on transfer fees by excluding them from the copy, like so:

```
~$ mkdir -p data/5HT2A/H17
~$ aws s3 cp --recursive --exclude "*OUTDOCK.gz*" s3://btingletestbucket/docking_runs/5HT2A/H17 data/5HT2A/H17
(lots of copy logs)
~$ ls data/5HT2A/H17
aa   ab
```

Step 4: Run Analysis and save results

All that's left to do is run analysis using the new top_poses.py script.

```
python3.8 ~/DOCK/ucsfdock/analysis/top_poses/top_poses.py ~/data/5HT2A/H17 -n 10000 -o ~/data/5HT2A/poses_H17 -j 1
```

This command, for example fetches the top 10,000 scoring molecules found in test.mol2.gz* files underneath the ~/data/5HT2A/poses_H17 directory on one core. See the linked wiki page for more details.

There is no need to provide a dirlist, top_poses.py will find all test.mol2* files beneath the directory you specify. Alternatively you can provide a text file containing a list of test.mol2.gz* files to analyze.

Two files will be created, a .mol2.gz file and a .scores file. The .mol2.gz file will contain the mol2 data for all top poses, and .scores will contain a text line for each top pose describing energy etc parameters.

You can copy these output files to your S3 bucket, or use SCP to copy them to your computer.

```
scp -i /path/to/my/key.pem ec2-user@XXX.XXX.XXX.XXX:~/data/5HT2A/H17/poses_H17.mol2.gz poses_H17.mol2.gz
```

IMPORTANT:

Once you're done, make sure to terminate the instance in your EC2 console. Be aware that any data on your instance will be deleted when you do this, so copy back anything you care about first.

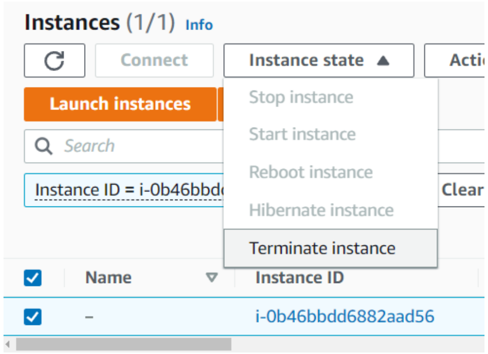

Retrieved from "http://wiki.docking.org/index.php?title=AWS:Merge_and_download_results&oldid=14796"

This page was last edited on 2022-09-14, at 14:30:00.
