# Supplementary material for: Large-Scale Docking in the Cloud
Source: J Chem Inf Model. 2023 Apr 18;63(9):2735–41. doi: 10.1021/acs.jcim.3c00031 (PMC10170500; doi:10.1021/acs.jcim.3c00031)
Supplement: Supplementary file 6 — ci3c00031_si_006.pdf [file ci3c00031_si_006.pdf]

AWS:Cleanup

- Tutorial 1: AWS:Set up account
- Tutorial 2: AWS:Upload files for docking
- Tutorial 3: AWS:Submit docking job
- Tutorial 4: AWS:Merge and download results
- Tutorial 5: AWS:Cleanup THIS TUTORIAL

This page is about how to clean up your AWS account after you have finished docking. Recall that you are likely going to want to return here in a 8-10 weeks when you have purchased compounds and tested them and you want to do some follow up docking.

Contents

option 1
option 2
option 3
option A

option 1

Leave everything as is. Advantage. you lose nothing. Disadvantage: you pay for storage

option 2

Delete the raw output in S3, retaining only the merged and sorted results and the overall set up with dockfiles and so on. Advantage: easy to resume calculations. Disadvantages: few. You wont have every score of every molecule, so you might have to re-dock some tranches if you want to look at that.

option 3

Delete everything from AWS S3, after downloading critical data to your own local host. Advantage: minimal monthly storage costs. Disadvantage. You would need to re-sync the data to resume docking.

option A

Set the storage option to automatic/glacier. Advantage: This will reduce your long-term storage costs. Disadvantage: you may have to wait hours to access your files. This is compatible with all options above.

Retrieved from "http://wiki.docking.org/index.php?title=AWS:Cleanup&oldid=14795"

This page was last edited on 2022-09-14, at 14:29:10.
